# The Effect of Tethers on Artificial Cell Membranes: A Coarse-Grained Molecular Dynamics Study

**DOI:** 10.1371/journal.pone.0162790

**Published:** 2016-10-13

**Authors:** William Hoiles, Rini Gupta, Bruce Cornell, Charles Cranfield, Vikram Krishnamurthy

**Affiliations:** 1 Department of Electrical and Computer Engineering, The University of British Columbia, Vancouver, British Columbia, Canada; 2 Director of Science and Technology, Surgical Diagnostics Pty Ltd., Unit 6 30-32 Barcoo Street, Roseville, New South Wales, 2069, Australia; 3 School of Life Sciences, University of Technology Sydney, Broadway, New South Wales, Australia; 4 Electrical and Computer Engineering, Cornell University, New York, New York, United States of America; University of Lincoln, UNITED KINGDOM

## Abstract

Tethered bilayer lipid membranes (tBLMs) provide a stable platform for modeling the dynamics and order of biological membranes where the tethers mimic the cytoskeletal supports present in biological cell membranes. In this paper coarse-grained molecular dynamics (CGMD) is applied to study the effects of tethers on lipid membrane properties. Using results from the CGMD model and the overdamped Fokker-Planck equation, we show that the diffusion tensor and particle density of water in the tBLM is spatially dependent. Further, it is shown that the membrane thickness, lipid diffusion, defect density, free energy of lipid flip-flop, and membrane dielectric permittivity are all dependent on the tether density. The numerically computed results from the CGMD model are in agreement with the experimentally measured results from tBLMs containing different tether densities and lipids derived from Archaebacteria. Additionally, using experimental measurements from Escherichia coli bacteria and Saccharomyces Cerevisiae yeast tethered membranes, we illustrate how previous molecular dynamics results can be combined with the proposed model to estimate the dielectric permittivity and defect density of these membranes as a function of tether density.

## Introduction

Biological membranes are essential for sensing and signalling in biology and act to separate the intracellular and extracellular domain of the cell. The complexity associated with studying biological membranes has driven studies of well defined synthetic bilayer lipid membranes. These include black lipid membranes [1, 2], vesicles [3], and supported bilayers [4, 5]. A new class of experimental work has focused on the use of tethered bilayer lipid membranes (tBLMs) which include tethers that mimic the physiological response of the cytoskeletal networks found in all biological cell membranes [6, 7]. A classic example is the Spectrin-Ankyrin–Band 3 network in Red Blood Cell (RBC) membranes. The Spectrin cytoskeletin is tethered to the RBC membrane via the linker protein Ankyrin, and subsequently to the Band 3 protein that traverses the RBC membrane. RBCs survive in the human body for over 120 days and undergo multiple changes in geometry as the cells pass through the heart and major blood vesicles and circulate through the microcapilliaries requiring thousands of alterations in aspect ratio for each passage around the body. Useful features of the tBLMs for experimentalists are that they are stable over weeks to months, are solvent free and are sufficiently distant from their solid supporting substrate due to the tether groups that they are not distorted by adsorption to the substrate. Their geometry permits convenient electrical access to both side of the membrane and measure by electrical impedance spectroscopy of the bilayer conductance and capacitance. In addition the stabilizing tethers mimic the behavior of the cytoskeletal supports providing one of the best models for studying the dynamics and order in biological membranes. The key question we focus on in this paper is how does the gold bioelectronic interface and the tether density impact membrane dynamics and biomechanics.

Models of tethered membranes have been reported using coarse-grained molecular dynamics (CGMD) [8, 9]. CGMD models provide reasonable accuracy given the current computational efficiency available on supercomputing clusters. In [8] the authors study properties associated with DOPC membranes tethered to a gold surface via polyethylene glycol chains. It was shown that diffusivity of lipids in the distal layer (adjacent to the electrolyte bath) are larger than the proximal layer (adjacent to the bioelectronic interface). This result is not unexpected as the tethered lipids impede the movement of lipids in the proximal layer. The results in [8] suggest that to minimize the number of membrane defects, a tether with 5-10 polyethylene glycol moieties and tether density below 22% should be used. For longer tethers and higher tether density the membrane undulates, forms significant defects, and dissociates from the surface. However, experimental measurements from tBLMs containing up to 11 polyethylene glycol moieties do not contain significant defects and have a lifetime of several weeks [10]. Using the CGMD model and experimental measurements from tethered bilayer lipid membranes, we show that for an increase in tether density the membrane stabilizes–that is, there is a reduction in the number of membrane defects as the tether density increases from 1% to 100%. In [9] the formation dynamics of a DPPC membrane tethered to gold via polyethylene glycol chains is presented. The lipid concentration selected in the formation process is critical to minimize the formation of defects in the membrane (if the concentration is too low), or the formation of micelles on the membrane surface (if the concentration is too high). Though [8, 9] provide valuable insight into tethered membrane dynamics, several questions remain. How does the tethering architecture (i.e. bioelectronic interface) impact the tethered membrane dynamics? What effect do different lipids have on the tethered membrane stability? Additionally what effect does the tether density (i.e. cytoskeletal support) have on the stability of membranes?

In this paper a CGMD model of a tethered archaebacterial membrane is generated based on the MARTINI force field [11, 12]. The CGMD model is used to gain key insights into the diffusion of water and lipids, membrane thickness, defect density, and the free energy of lipid flip-flop as a function of tether density. The numerically computed results from the CGMD model are in agreement with the results from experimental measurements from tethered bilayer lipid membranes. Using the results from experimental measurements we show that a combination of diffusion-limited charge transfer, and ionic adsorption is present at the bioelectronic interface. These double-layer charging effects can be modeled using fractional order operators [13]. It is shown that the dielectric permittivity of tethered membranes is dependent on the tether density. To investigate the effect tethers and lipid architecture have on membrane stability, we construct tethered bilayer lipid membranes composed of archaebacterial lipids, and lipids from E. coli and Saccharomyces cerevisiae (S. cerevisiae) membranes. We show that archaebacterial lipids are the most resistant to electroporation, with E. coli having a higher resistance compared to lipids from S. cerevisiae.

## Methods

### Coarse-Grained Molecular Dynamics Model of Tethered Bilayer Lipid Membrane

The coarse-grained molecular dynamics model of the tethered bilayer lipid membrane is constructed using the MARTINI force field [11, 12]. Parameters modeled include the spatially dependent diffusion tensor **D**, the thickness of the membrane *h*_*m*_, the surface tension *σ*, and the line tension *γ*, defect density, and free energy of lipid flip-flop which are all dependent on the tethering density.

**Fig 1:**
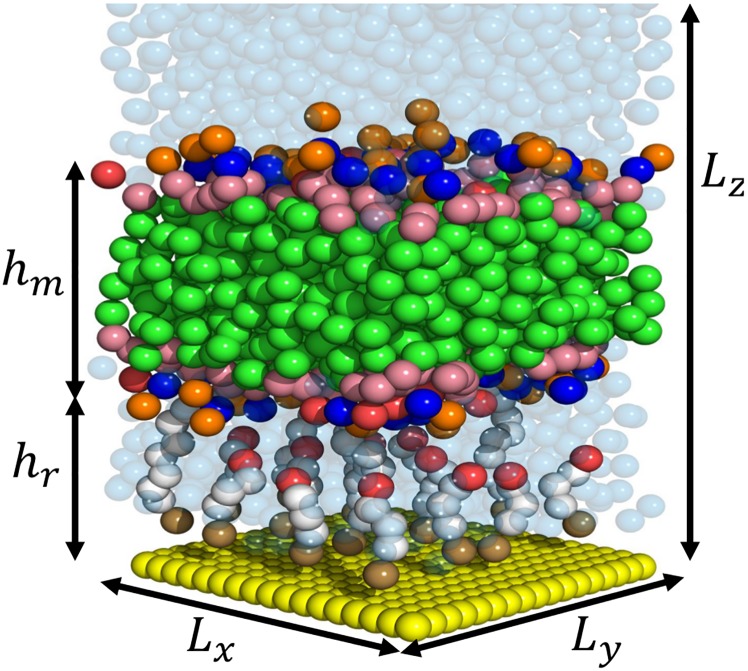
Schematic of the CGMD model of the tethered bilayer lipid membrane. The green beads represent the phytanyl tail of the GDPE and DphPC lipids, NC_3_ bead is displayed in blue, the PO_4_ bead in orange, OH bead in red, the COC bead as pink, polyethylene glycol beads as white, benzyl disulphide bead as brown, gold beads as yellow, and the water beads as a translucent blue. *L*_*z*_ is the height of the simulation cell, *L*_*y*_ and *L*_*x*_ are the length and width of the simulation cell, *h*_*r*_ is the tethering reservoir height, and *h*_*m*_ is the membrane thickness.

To construct the CGMD model the molecular components of the tethered bilayer lipid membrane are mapped into the MARTINI [11, 12] force field. The molecular components include the zwittrionic C20 diphytanyl-ether-glycero-phosphatidylcholine lipid (DphPC), C20 diphytanyl-diglyceride ether lipid (GDPE), benzyl disulphide connected to an eight-oxygen-ethylene-glycol group terminated by a C20 hydrophobic phytanyl chain (tether), benzyl disulphide connected to a four-oxygen-ethylene-glycol group terminated by an OH (spacer), and the gold surface. The mobile lipid phase is physically tethered to the chemically attached layer is composed of a 30% GDPE to 70% DphPC lipid membrane. To explore the effects of tether density on the dynamics and stability of the tethered membrane, 0% and 25% tether density (i.e. a 25% area tether density reflects one spacer molecular for every three tethering molecules) CGMD models of the tethered membrane are constructed. The CGMD model of the 25% tethered membrane is provided in Fig 1. A detailed description of the 0% and 25% CGMD models is provided below. Note that unless otherwise specified, the bead types and interactions are provided in [11, 12].

**Lipids:** The phosphatidylcholine headgroup of the DphPC lipid is represented by two beads: the positive choline by the Q_*o*_ bead, and the negative phosphate by the Q_*a*_ bead. The ether-glycol is represented by a SN_*a*_ bead, and each of the phytanyl tails by four C_1_ beads. The phytanyl and ether glycerol moieties of GDPE are represented by the same mapping as the DphPC, however the hydroxyl headgroup of GDPE is represented by a P_4_ bead. In total the DphPC lipid is composed of 12 coarse-grain beads, and the GDPE lipid by 11 coarse-grain beads.

**Tethers and Spacers:** The eight ethylene glycol molecules of the tethers are represented by 8 PEG beads. The interaction of the PEG beads is provided in [9, 14]. The benzyl disulphide group is represented by a C_5_ bead which has the highest polar affinity of the MARTINI coarse-grain beads [11]. The phytanyl tail of the tethers is represented by four C_1_ beads. The spacers are mapped using an identical method as the tethers however the hydroxyl group is represented by the P_4_ bead.

**Gold Surface:** The gold surface is composed of a square lattice with custom P_*f*_ beads spaced 0.3 nm apart. The interaction between P_*f*_ and P_4_ is 1/3 the value between P_4_ and P_4_, and the interaction between P_*f*_ and other bead types is ∼12% of the MARTINI value between P_4_ and respective bead types. The following interactions are excluded: interaction between P_*f*_ beads, and between the C_5_ beads of the tethers and spacers, and *P*_4_ and Q_*o*_ beads of the lipids. The interactions and spacing of the P_*f*_ beads are selected to reduce the effects of excess adsorption of beads to the surface–that is, the surface mimics a hydrophobic gold surface. Note that from the ab initio molecular dynamics results in [15], there is a wetting layer of water at the gold surface, however beyond 3.2 Å one of the hydrogen molecules in water points towards the gold surface which is typical for many hydrophobic surfaces. Given that in the CGMD model each water bead represents four water molecules, the water bead interaction with the gold bead surface is selected to be hydrophobic.

### Diffusion Tensor from Coarse-Grained Molecular Dynamics Model

The simulation results from the CGMD model are the trajectories of coarse-grained beads as a function of time. In this section a method is provided to compute the spatially dependent diffusion tensor from the CGMD trajectories.

To estimate the diffusion tensor **D** of species from CGMD trajectories requires a probabilistic model. Let Ψ denote the conditional probability density function of observing a bead at position *x* at time *t* that was initially at position *x*_*o*_ at time *t*_*o*_. Assuming the bead’s dynamics satisfy a Weiner process, then the time evolution of *Ψ* is given by the overdamped Fokker-Planck equation [16]:

$$\begin{array}{c} \frac{\partial \Psi }{\partial t}=\nabla \cdot D\cdot \left[\nabla +\beta \left(\nabla F_{w}\right)\right]\Psi . \end{array}$$ (1)

In Eq (1), **D** is the diffusion tensor, *F* the free energy, and *β* the inverse of the thermal free energy (i.e. *β* = 1/*k*_*B*_ *T*). Consider the CGMD model of the tethered membrane in Fig 1. The boundary conditions of Eq (1) in *z* are composed of no-flux boundary conditions at the gold bioelectronic interface, denoted by *z*_*b*_, and the surface of the tethered membrane, denoted by *z*_*t*_, with the others defined as infinite boundary conditions. From the coordinate axis of the CGMD model, refer to Fig 1, the diffusion tensor is diagonal with *D*_*xx*_ = *D*_*yy*_ ≠ *D*_*zz*_. The translational invariance parallel to the confining surface ensures that *F*_*w*_ in the parallel direction is constant (i.e. *F*_*w*_ is invariant in *x* and *y*). The free energy of water along the *z*-axis is related to the equilibrium density of water by *F*_*w*_(*z*) = −*k*_*B*_ *T* log(*ρ*(*z*)/*ρ*_*o*_) with *ρ*_*o*_ the bulk density. As a result of the no-flux boundary conditions in *z*, the diffusion coefficients in **D**, defined below Eq (1), will be a function of *z*. If we discretize the *z*-dimension sufficiently into layers, then in each layer the diffusion coefficients will not vary substantially. This allows Ψ Eq (1) to be decoupled such that Ψ = Ψ_*x*_ Ψ_*y*_ Ψ_*z*_ with Ψ_*x*_(*x*, *t*|*x*_*o*_, *t*_*o*_), Ψ_*y*_(*y*, *t*|*y*_*o*_, *t*_*o*_), Ψ_*z*_(*z*, *t*|*z*_*o*_, *t*_*o*_) denoting the time evolution of the particle in each respective dimension.

The partial differential equation modeling the time evolution of Ψ_*x*_(*x*, *t*|*x*_*o*_, *t*_*o*_) = Ψ_*y*_(*y*, *t*|*y*_*o*_, *t*_*o*_) is given by Fick’s second law of diffusion. For an infinite boundary condition, the analytical solution of Ψ_*x*_ and Ψ_*y*_ is given in terms of the Green’s function. This allows *D*_*xx*_ = *D*_*yy*_ to be estimated by the evaluation of the second central moment (i.e. for *D*_*xx*_ the relation 〈(*x* − *x*_*o*_)^2^〉 = 2*D*_*xx*_(*t* − *t*_*o*_) holds with the ensemble average taken over time). The time evolution of Ψ_*z*_ is given by:

$$\begin{array}{c} \frac{\partial \Psi_{z}}{\partial t}=\frac{\partial }{\partial z}\left[D_{zz}e^{-\beta F(z)}\frac{\partial }{\partial z}\left(\Psi_{z}e^{\beta F(z)}\right)\right]. \end{array}$$ (2)

No analytical solution exists for Eq (2) given the no-flux boundary conditions at *z*_*b*_ and *z*_*t*_. If we consider the round-trip time, denoted by *τ*_*rt*_, then *D*_*zz*_ can be evaluated using [17]:

$$\begin{array}{c} D_{zz}\left(z\right)=\frac{e^{\beta F(z)}}{\partial \tau_{rt}\left(z\right)/\partial z}\int_{z_{b}}^{z_{t}}e^{-\beta F(z^{\prime })}dz^{\prime }. \end{array}$$ (3)

In Eq (3), *τ*_*rt*_ denotes the time needed to start at *z*, reach a position z_*_, start from z_*_ again, and reach *z* (i.e. the round-trip time). The estimation of *τ*_*rt*_ can be obtained directly from the CGMD trajectories allowing Eq (3) to be used to estimate *D*_*zz*_(*z*).

### Coarse-Grained Molecular Dynamics Simulation Details

The coarse-grained molecular dynamics simulations were performed using GROMACS [18] version 4.6.2 (double precision) with the MARTINI force field [11, 12]. Unless otherwise stated, the CGMD simulation parameters are provided in [8, 9]. The interaction of the CGMD beads are defined by the Lennard-Jones (LJ) potential, and harmonic potentials are utilized for bond and angle interactions. A shift function is added to the Coulombic force to smoothly and continuously decay to zero from 0 nm to 1.2 nm. The LJ interactions were treated likewise except that the shift function was turned on between 0.9 nm and 1.2 nm. The grid-type neighbour searching algorithm is utilized for the simulation–that is, atoms in the neighbouring grid were updated every ten time steps. The equations of motion are integrated using the leapfrog algorithm with a timestep of 20 fs. The temperature is held constant at 320 K using a velocity rescaling algorithm [19] with a time constant of 0.5 ps. The lipids, tethers, spacers and water molecules are coupled separately for temperature control. Production runs are performed in the NVT ensemble using periodic boundary conditions in three-dimensions with a total simulation time horizon of 1.5 *μ*s. Visualization of the results are reported using VMD and PyMOL. The numerical method used to evaluate *D*_*zz*_ Eq (3) is provided in [20].

Prior to the final NVT production we first equilibrate the CGMD system sufficiently to model the experimental conditions. Currently there are no methods to perform an NPT ensemble simulation for CGMD systems that contain a substrate as the interaction site density of the surface must be kept constant–equivalently, it is desirable to keep the interaction strength of the substrate constant [8, 21–23]. Therefore, either the NVT or NAP_z_T ensembles are used to perform simulations with a substrate surfaces present [8, 21–23]. To minimize the induced pressure effects caused by the constant area constraint, the following steps are performed prior to the NVT production runs. First, the membrane and solvent, with no substrate, are equilibrated for 50 ns using the steepest descent method in GROMACS. Then a production run in the NPT ensemble is performed for 1 *μ*s to ensure that the membrane and solvent reach a sufficiently equilibrated pressure of 1 bar. Second, the substrate is added to the top and bottom of the simulation cell. The gold substrate area and particle density are chosen as a best compromise between the equilibrium area per head-group of a free-standing DphPC bilayer (69 Å) and the commensurability with the gold substrate surface. The initial dimensions of the simulation cell are *L*_*x*_ = *L*_*y*_ = 10.8 nm, and *L*_*z*_ = 15.2 nm. Another 1 *μ*s production run is performed using the NAP_z_T ensemble ensuring the substrate area is constant while allowing the normal pressure to fluctuate around the reference value of 1 bar. The pressure is coupled semi-isotropically in the NAP_z_T with P_z_ coupled to 1 bar by the Berendsen barostat using *τ* = 5.0 ps and a compressibility of 3 × 10^−5^ bar^−1^. After the 1 *μ*s NAP_z_T ensemble simulation the final simulation cell size is *L*_*x*_ = *L*_*y*_ = 10.8 nm, and *L*_*z*_ = 16.0 nm. An NVT ensemble with these simulation cell dimensions is used for all subsequent production runs.

Is the simulation cell size for the NVT ensemble sufficiently large to accurately estimate the lateral diffusion coefficient of the lipids? The simulation cell size should be sufficiently large to reduce the effects caused by highly correlated transitions of lipids. This results as the movement of one lipid perturbs its neighbours and propagates across the periodic boundaries resulting in highly correlated lipid movements. Generally this effect is not present for system that contain over 100 lipid molecules [24–26]. However, currently there are no theoretical guarantees of simulation cell sizes that can eliminate this correlation effect. For example, it may be the case that lipids undergo collective diffusion on length scales ranging from a few to several tens of nanometers [27–31]. To minimize the probability of such an effect while still ensuring computational tractability we use a simulation cell size of *L*_*x*_ = *L*_*y*_ = 10.8 nm, and *L*_*z*_ = 16.0 nm which contains 320 lipid molecules. Assuming that the Saffman-Delbrück hydrodynamic model is valid (e.g. assuming no tethers, mean-field approximations hold, and the elementary model of a bilayer [32] hold), the estimated lateral diffusion coefficient with this simulation cell size should be approximately equal to the actual diffusion coefficient with a maximum error of 10% to 20% [28].

### Tethered Bilayer Lipid Membrane Preparation

To validate the results from the CGMD model and to better understand the effect of tethers and the solid gold bioelectronic interface have on the membrane dynamics and order, we utilize experimental measurements from tethered bilayer lipid membranes. The fabrication procedure of the tethered membrane id described below. In the S1 Text a fractional order macroscopic model is provided to permit the use of the results from experimental measurements to estimate important membrane properties (these include line tension, surface tension, membrane capacitance, and membrane conductance).

The tethered membrane is supported by a 25 × 75 × 1 mm polycarbonate slide. Six 100 nm thick sputtered gold electrodes, each with dimensions 0.7 × 3 mm, are patterned on the polycarbonate slide. Each electrode is contained within an isolated flow cell with a common opposing, large area gold return electrode. The formation of the tethered membrane is performed in two stages using the solvent-exchange technique presented in [7]. The first stage of formation involves anchoring of the tethers and spacers to the gold surface. The tethers provide structural integrity to the membrane and mimic the cytoskeletal supports of natural cell membranes. The spacers laterally separate the tethers allowing patches of mobile lipids to diffuse in the membrane. The spacer is composed of a benzyl disulphide connected to a four-oxygen-ethylene-glycol group terminated by an OH; the tethers are composed of a benzyl disulphide connected to an eight-oxygen-ethylene-glycol group terminated by a C20 hydrophobic phytanyl chain. To form the anchoring layer, an ethanolic solution containing 370 *μ*M of engineered ratios of benzyl disulphide components is prepared. This solution is exposed to the gold surface for 1 to 2 hours, then the surface is flushed with ethanol and air dried for approximately 2 min. Note that in the special case of 100% tethering, the engineered tethered membrane is composed of a tethered monolayer with no spacer molecules. The second stage is the formation of the tethered membrane. The mobile lipids are composed of synthetic mixtures of zwittrionic C20 diphytanyl-either-glycero-phosphatidylcholine lipid (DphPC), and C20 diphytanyl-diglyceride either (GDPE). The solution containing the mixture of mobile lipids is added to the gold bonded components from the first stage. Several lipid solvents can be used [7, 33], however in most cases the lipids selected to form the bilayer are soluble in ethanol. As an example, 8 *μ*L of 3 mM mixture of 70% DphPC and 30% GDPE in ethanol is added to the flow cell chamber containing the gold bonded layer. The solution is incubated for 2 min at 20°C in which the tethered membrane forms. Following a 1.5 to 2 min incubation, 300 *μ*L of phosphate buffered saline is flushed through each flow cell chamber. The tethered membrane is equilibrated for 30 min prior to performing any experimental measurements. The formation of the E. coli, and S. caerevisae tethered membranes follows a similar procedure.

All experimental measurements were conducted at 20°C in a phosphate buffered solution with a pH of 7.2, and a 0.15 M saline solution composed of Na^+^, K^+^, and Cl^−^. At this temperature the tethered membrane is in the liquid phase. A pH of 7.2 was selected to match that typically found in the cellular crystalline fluid phase. The quality of the tethered membrane is measured continuously using an SDx tethered membranes tethaPod^™^ swept frequency impedance reader operating at frequencies of 1000, 500, 200, 100, 40, 20, 10, 5, 2, 1, 0.5, 0.1 Hz and an excitation potential of 25 mV (SDx Tethered Membranes, Roseville, 2069, NSW, Sydney, Australia). Refer to the S1 Fig for the associated impedance measurements. Custom drive potentials are produced and the resulting current recorded using an eDAQ^™^ ER466 potentiostat (eDAQ, Doig Ave., Denistone East) and a SDx tethered membrane tethaPlate^™^ adaptor to connect to the assembled electrode and cartridge.

## Results

### Order and Dynamics of Tethered Bilayer Lipid Membranes

The structure and order of the tethered bilayer lipid membranes (tBLMs) is modelled using simulation results from the CGMD model (Fig 1). Computed properties include the spatially dependent density and diffusion tensor **D**, membrane thickness *h*_*m*_, surface tension *σ*, and line tension *γ* are computed.

**Fig 2:**
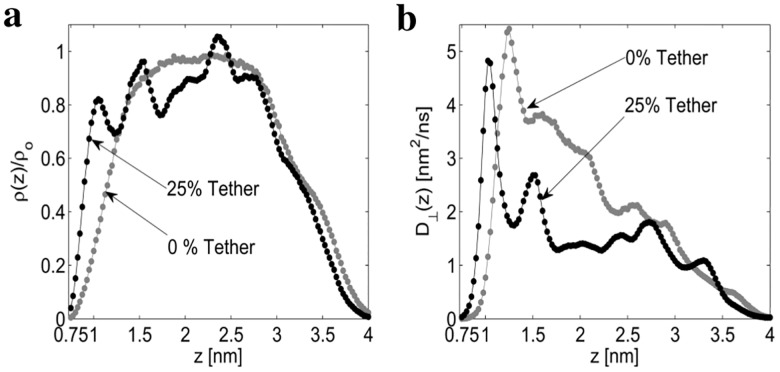
Normalized water density and perpendicular diffusion *D*_⊥_(*z*). The normalized density *ρ*(*z*)/*ρ*_*o*_ (a) and perpendicular diffusion coefficient *D*_⊥_(*z*) (b) are computed from the CGMD bead trajectories.

The density of water for the 0% and 25% tethered membranes is provided in Fig 2a. As expected, the density of water is attenuated at the gold interface and membrane interface for both the 0% and 25% tethered membranes. However, by comparing the density of the 0% and 25% tethered membranes, it is apparent that the density of water is dependent on the tether density. Given the water density is position-dependent, this strongly suggests the self-diffusion tensor of the water will also be position dependent. Using Eq (3), the position dependent perpendicular diffusion coefficient *D*_⊥_(*z*) is provided in Fig 2b. From Eq (3), the diffusion coefficient *D*_⊥_(*z*) is dependent on the round-trip time *τ*_*rt*_(*z*) and the density profile *ρ*(*z*). Intuitively, the slope of the round-trip time *τ*_*rt*_(*z*) is inversely proportional to *D*_⊥_(*z*). In Fig 2b the initial peak at 1.05 nm and second peak at 1.52 nm of the 25% diffusion profile match the location of the density peaks at 1.05 nm and 1.52 nm in Fig 2a. The initial peak at 1.25 nm for the 0% diffusion profile results as the round-trip time of a water bead in this region is approximately constant. Additionally, for the 0% tethered membrane, the density profile remains approximately constant for *z* = 2.0 nm to *z* = 2.5, however the round-trip time (S2 Fig) does not remain constant. The combined effect of the constant density but varying round-trip time causes *D*_⊥_(*z*) to be non-constant in this region. In the (S3 Fig) the water density and spatially dependent diffusion coefficient for the gold interface with no membrane present is provided. From the (S3 Fig), the diffusion coefficient *D*_⊥_(*z*) approximately reaches the bulk diffusion coefficient for *z* ≥ 2.5 nm from the gold interface with no membrane present.

The computed lateral diffusion of the DphPC lipids, GDPE lipids, and water for the 0% and 25% tethered membrane are provided in Table 1.

**Table 1:** Lateral Diffusion Coefficient *D*_||_ (nm^2^/*μ*s).

Tethering density		0%	25%
Proximal Layer	DphPC	290±23	87±10
	GDPE	256±36	116±16
Distal Layer	DphPC	289±24	115±12
	GDPE	256±38	128±13
Bulk Water		2105±288	2010±233
Tethering Reservoir Water		2730±258	1236±110

The computed lateral diffusion of the DphPC and GDPE lipids in the proximal (i.e. adjacent to the tethering reservoir) layer and distal (i.e. adjacent to the bulk water) are nearly identical. The diffusion of DphPC is related to that of GDPE by a multiplicative factor of 1.13. The effects of the tethers cause the lateral diffusion coefficient of DphPC to decrease by a factor of approximately 3.3 in the proximal layer, and 2.5 in the distal layer. Similarly for GDPE the decrease is 2.2 in the proximal layer, and 2.2 in the distal layer. This result is in agreement with the experimental results reported in [34, 35] for different lipids and tethering densities. The lateral diffusion of water in the bulk region is related to the tethering reservoir lateral diffusion by a factor of approximately 1.6 for the 25% tethered membrane. It is interesting that the lateral diffusion of water in the tethering reservoir is 2.7 nm^2^/ns which is close to but faster than that in the bulk of 2.1 nm^2^/ns for the 0% tethered membrane. The interplay between hydrogen bond breaking and cooperative rearrangement of regions of approximately 1 nm in size cause the lateral diffusion to significantly increase in nanoconfined water regions [36]. Since explicit hydrogen bonds are not included in the CGMD model [12], we attribute the increased diffusion coefficient to the cooperative rearrangement of water molecules. A unique result of this study is that no anomalous diffusion was detected for water and lipid headgroups near the outer surface of bilayer lipid membrane. This is in contrast to other dynamical studies [37–40] on different membranes which report an anomalous water and lipid diffusivity in the vicinity of the membrane surface.

To compute the membrane thickness *h*_*m*_ (Fig 1) the particle density of the lipid headgroups is used. The thickness of the 25% and 0% tethered membrane is *h*_*m*_ = 3.53 nm and *h*_*m*_ = 3.48 respectively. The thickness of the phytanyl tails (i.e. hydrocarbon tails) was also computed for the 25% and 0% tethered membranes and is 2.15 nm and 2.11 nm respectively. The reservoir thickness *h*_*r*_ (Fig 1) of the 25% and 0% tethered membrane is *h*_*r*_ = 3.30 nm respectively. These numerically computed values are consistent with the experimentally measured thickness for DphPC based tethered membranes [41].

To compute the line tension *γ*, and surface tension *σ* the method provided in [42] was utilized. The resulting values are: *σ* = 15 mN/m, and *γ* = 12 pN. These values are in agreement with the experimental results [43] and simulation results [44–46] reported in the literature for similar DphPC based membranes. Note that surface tension *σ* is a key mechanical property in regulating the motility and the reshaping of bilayer membranes. A bilayer membrane with no tethers simulated in a NPT ensemble will have zero surface tension *σ* = 0 mN/m as there is no induced stress on the membrane [47, 48]. However, if an external stimuli is applied then a non-zero surface tension will result [49–51]. The induced surface tension in a tethered membrane results from the pressure differences across the membrane surface, and the adhesion of the membrane to the tethered supports. Note that the induced surface tension from the tethers is analogous to the induced surface tension in cellular membranes from the cytoskeletal supports [52–54]. The computed surface tension of the tethered DphPC membrane is *σ* = 15 mN/m which indicates that the tethers introduce a non-zero stress on the DphPC membrane. As *σ* > 0 mN/m the tethers slightly increase the area-per-lipid from their equilibrium value–that is, the tethers cause a slight increase in DphPC membrane area compared to the untethered DphPC membrane area.

### Lipid Energetics and Defect Density

The potential of mean force (PMF) of lipids in a lipid bilayer is a key thermodynamic property that can be used to estimate the defect density and the free energy of lipid flip-flop. Lipid flip-flop occurs when a lipid molecule flips from one side of the lipid bilayer to the other. Here the umbrella sampling method [55] is used to compute the PMF for moving a single DphPC lipid along the normal to the membrane surface for both the 0% and 25% tethered membranes. The umbrella potential acts on the center of mass of the phosphate group of the DphPC lipid with a harmonic restraint with a force constant of 500 kJ/mol/nm^2^ in the direction normal to the membrane surface. The spacing between biasing potentials was selected as 0.15 nm and 0.1 nm for the 0% and 25% tethered membranes. This resulted in 37 and 48 parallel simulations for the 0% and 25% tethered membranes respectively. The starting structures corresponding to each umbrella window were created by pulling a lipid to their respective position using an umbrella potential with a force constant of 500 kJ/mol/nm^2^ in a 1 ns simulation. Each umbrella window was then equilibrated for 50 ns with a 1000 kJ/mol/nm^2^ force constant, followed by a 1 *μ*s simulation. The PMF profile was constructed from the biased distributions of the center of mass of the lipids using the weighted histogram analysis method [56] with a relative tolerance of 10^−4^.

**Fig 3:**
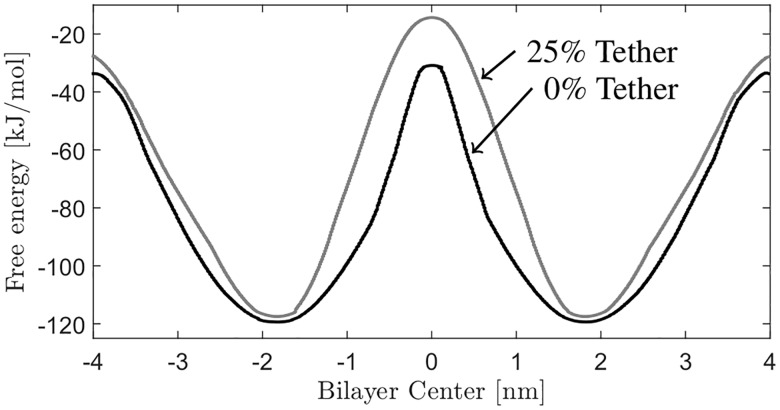
PMF of DphPC lipid. The PMFs for a 0% and 25% tether density DphPC lipid membrane.

The computed PMF for both tethering densities is provided in Fig 3. As expected, the minimum of the PMFs at -1.82 nm and 1.82 nm corresponds to the equilibrium position of the DphPC lipid. There is an increase in the PMF from these equilibrium positions as a result of the hydrophobic tail coming into contact with the water, and the hydrophilic headgroups coming into contact with the hydrophobic phytanyl tails of the adjacent lipids. The difference in energy between the equilibrium position and when fully solvated in the water solution corresponds to the free energy of lipid desorption. The free energy of desorption for the 0% and 25% membrane is 85.93 kJ/mol and 91 kJ/mol respectively. As expected, tethers cause the associated energy of lipid desorption to increase as compared with the untethered membrane. As the lipid is moved to the center of the membrane, the steep slope in the PMF is caused by the lipid headgroup interacting with water and the hydrophobic interior of the membrane interior. This characteristic of the PMF has been observed when other charged/polar molecules are transferred into the hydrophobic interior of the membrane and is associated with the formation of an aqueous pore (i.e. membrane defect) [57–60].

Using the PMF (Fig 3), the free energy required for complete flip-flop of a single lipid is equal to the energy required to move a lipid from it’s equilibrium position to the center of the bilayer, then to the other leaflet’s equilibrium position. Therefore, from the maxima in the PMF’s between the equilibrium position and the bilayer center, the free energy barrier for flip-flop of the DphPC lipid increases from 89 kJ/mol for the free bilayer to 103.17 kJ/mol for the 25% tethered membrane. Notice that the lipid flip-flop energy of the 0% membrane is higher then for DOPC (87 kJ/mol) [60], DPPC (78 kJ/mol), DMPC (45 kJ/mol), and DLPC (17 kJ/mol) [57] as expected because archaebacterial lipids have a higher stability compared to these lipids. Additionally comparing the lipid flip-flop energy between the 0% and 25% tethered membrane, these results show that inclusion of tethers in the DphPC membrane prevents the formation of defects and increases the free energy for the translocation of charged headgroups across the membrane.

How is the energetics of lipid flip-flop associated with the membrane conductance? Assuming that ions only cross the membrane interface via membrane defects, then as the membrane defect density increases it is expected that the associated membrane conductance will also increase. Using the methods presented in [57, 61], the defect density *ρ*0 = e^−*β*Δ*G*_*p*_^/*A*_*L*_, with Δ*G*_*p*_ the associated energy required to form a defect, can be estimated. As illustrated in [57, 61–64], and in agreement with our CGMD simulation results, when the lipid headgroup is positioned in the center of the membrane a membrane defect forms. Therefore, we associated the energy required for lipid flip-flop with the energy required to form a membrane defect. Additionally, as the membrane defect density increases the associated conductance of the membrane increases as more pathways for ion transport through the membrane are present [57, 61]. Given that as the tether density increases the associated defect density decreases, it is expected that the membrane conductance decreases for increasing tether density. This is in agreement with our experimental results which show that as the tether density increases the associated membrane conductance decreases.

### Measured Dynamics and Order of Tethered Bilayer Lipid Membranes

**Fig 4:**
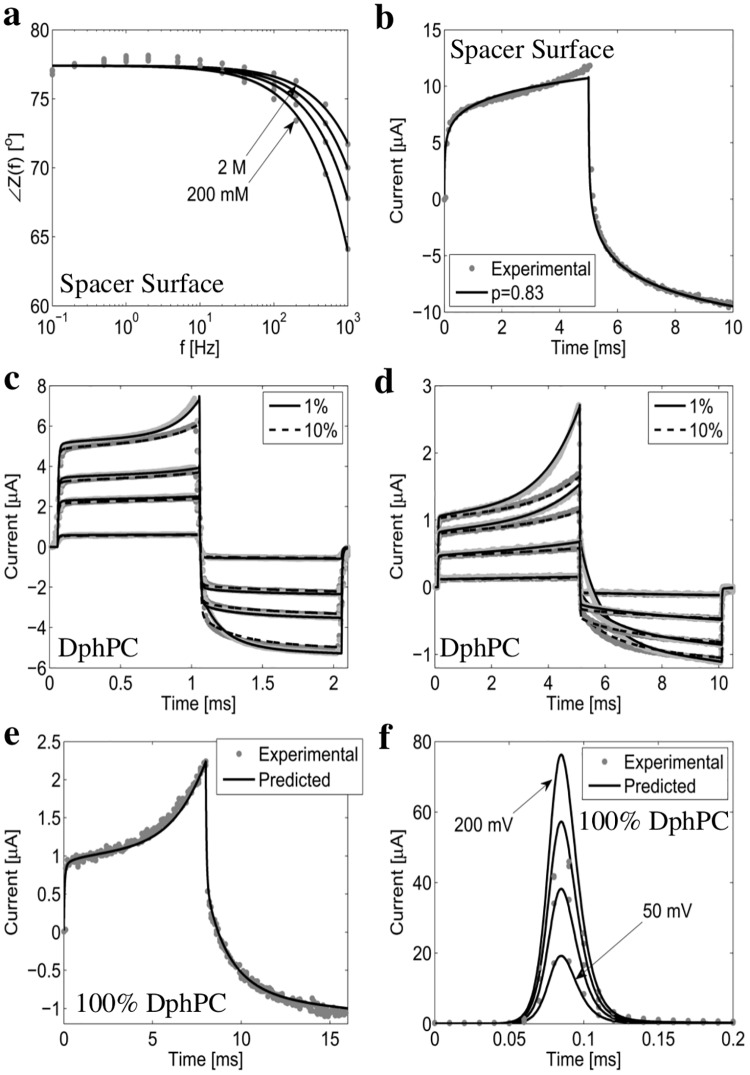
Experimentally measured and numerically computed current response of the tethered membrane. (a) and (b) provide the measured and computed phase and current response of the spacer surface. Note *C*_*m*_ → ∞ and *G*_*m*_ = 0 as the membrane is not present. (c) to (f) provide the experimentally measured and numerically computed current response *I*(*t*) for 1%, 10%, and 100% tethered DphPC membranes. In (c) the drive potential *V*_*s*_(*t*) is defined by a 1 ms linearly increasing potential with a slope of 10 V/s, 40 V/s, 70 V/s, and 90 V/s followed by a 1 ms linearly decreasing potential with identical slope. In (d) *V*_*s*_(*t*) is a 5 ms linearly increasing potential with slope of 50 V/s, 200 V/s, 300 V/s, and 450 V/s followed by a 5 ms linearly decreasing potential with identical slope. In (e) *V*_*s*_(*t*) is defined by a 8 ms linearly increasing potential with a slope of 100 V/s followed by a 8 ms decreasing potential with identical slope. In (f) *V*_*s*_(*t*) is a step of 50 mV, 100 mV, 150 mV, and 200 mV. All numerical results are computed using the fractional order macroscopic model provided in the S1 Text.

The surface of the gold electrode anchors the self-assembled monolayer of both the spacer and tether molecules. To investigate the effect of this surface on diffusion processes within the tBLM, an experimental test element was constructed employing only spacer molecules attached to the gold surface, denoted as the spacer surface. The phase behavior of ions charging and discharging the spacer surface for electrolyte concentrations of 2 M, 1 M, 500 mM, 200 mM NaCl is experimentally measured and modeled numerically. Results are given in Fig 4a. In the absence of a diffusion limited process ∠*Z*(*f*) = 90° for *f* < 1 Hz where the capacitive effects of the bioelectronic interface are dominant. As seen from Fig 4a, for *f* < 1 Hz ∠*Z*(*f*) = 76°, therefore for all concentrations measured there is a diffusion-limited process present with a fractional order parameter *p* = 0.86. To illustrate the accuracy of the fractional order macroscopic model (S1 Text), Fig 4b provides the experimentally measured and numerically modeled current response of the spacer only electrode for a drive potential *V*_*s*_ defined by a 100 V/s rise for 5 ms, and a -100 V/s fall for 5 ms. As seen, the fractional-order differential equation fractional order operator *p* = 0.83 provides an accurate prediction of the current response. The fractional-order behavior at the electrode surface may be caused by: diffusion-limited charge transfer and adsorption on the electrode [38]. Though the source of the behavior is unknown, the dynamics of the interface can be modeled using the fractional order differential equation (S1 Text) as illustrated in Fig 4a and 4b.

The tether density would be anticipated to impact membrane stability. As suggested from the CGMD results, the higher the tethering density the more resistant the membrane is to membrane defects. To validate this result we have fabricated tethered membranes with different tethering densities from 1%, 10%, and 100% and applied different transmembrane potentials to observe the dynamics of the defect density. Fig 4c to 4f show the results for 1%, 10%, and 100% tethering densities and various drive potentials. As expected, negligible defects are present for drive potentials below 250 mV as seen in Fig 4c and 4d. For larger drive potentials the formation of defects becomes pronounced, as seen in Fig 4c and 4d. It was found that the defect density in the 10% tethered membrane was always less than in the 1% tethered membrane, in agreement with the CGMD results. In the limiting case of a tethered membrane with 100% tether density, it was anticipated they would be the most resistant to membrane defects. Fig 4e shows that the 100% tethered membrane can withstand drive potentials of up to 800 mV. To determine whether the membrane was irreversibly damaged, the current response was compared to that of the numerical model for potential steps of 50 mV, 100 mV, 150 mV, and 200 mV. Fig 4f shows excellent agreement between the experimentally measured current and numerically computed current suggesting all current pathways formed by the voltage gradient reseal.

The above analysis shows that there exists a diffusion-limited process at the bioelectronic gold interface of tethered membranes, and that as the tethering density increases the number of membrane defects decreases.

### Effect of Variations in Membrane Composition and Tether Density

**Fig 5:**
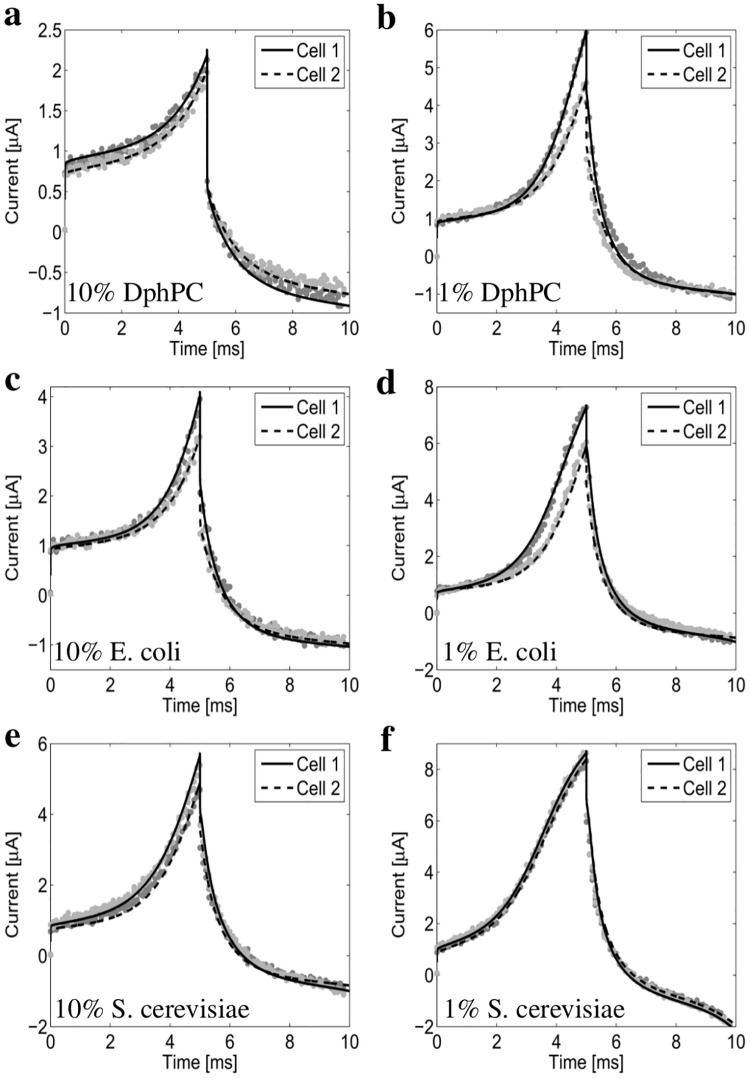
The measured and numerically computed current response of the 1% and 10% tether density DphPC, S. cerevisiae, and E. coli membranes. The excitation potential *V*_*s*_ is defined by a linear ramp of 100 V/s for 5 ms followed by a -100 V/s for 5 ms. Cell 1 and Cell 2 denote tethered membranes prepared using an identical procedure. All numerical results are computed using the fractional order macroscopic model provided in the S1 Text.

In this section the results from the CGMD model and experimental measurements are utilized to gain insight into important biological parameters as a function of tether density and lipid composition. The results presented are in agreement with the molecular dynamics results reported in [65, 66]. We consider tethered membranes composed of DphPC, S. cerevisae, and E. coli lipids with a 1% and 10% density are constructed. Fig 5 provides the numerically computed and experimentally measured current response. As seen, excellent agreement is obtained between the computed and measured current response. This allows the fractional order macroscopic model, provided in S1, and the CGMD model to be used to estimate important biological parameters.

Consider the relative dielectric permittivity *ε*_*m*_ of the tethered DphPC, E. Coli, and S. cerevisiae membranes. For homogeneous hydrocarbon tails the permittivity is approximately 2, however the lipid headgroups cause the effective permittivity *ε*_*m*_ to be larger. For the 10% tethered DphPC membrane *h*_*m*_ = 3.5 nm, *A*_*m*_ = 2.1 mm^2^, and *C*_*m*_ is the range of 12.5 nF to 15.5 nF. Therefore the relative permittivity is *ε*_*m*_ = 2.35 − 2.92. For the 1% tethered DphPC *C*_*m*_ is in the range of 16 nF to 17.5 nF, and assuming the thickness of identical to that of the 0% tethered DphPC *h*_*m*_ = 3.4 nm, the relative permittivity is *ε*_*m*_ is in the range 2.92 to 3.20. Note that the relative permittivity of the membrane *ε*_*m*_ must be between the values of 2 for pure hydrocarbon and 80 for an electrolyte at physiological concentrations. Therefore as the density of water increases between the distal and proximal layer, or the hydrocarbon thickness decreases it is expected that the relative permittivity will increase. Using the CGMD results in the Structure and Biomechanics section the decrease in *h*_*m*_ between the tethered and untethered DphPC membrane is a result of changes in the thickness of the hydrocarbon region. Therefore the decrease in *ε*_*m*_ between the 10% and 1% tethered DphPC membranes results from changes in the hydrocarbon thickness. This shows that the dielectric permittivity is dependent on the tether density. To estimate the permittivity of the tethered E. coli and S. cerevisae membranes we chose *h*_*m*_ = 3.29 nm for the E. coli, and *h*_*m*_ = 4.30 nm for the S. cerevisae as justified by the molecular dynamics results [66, 67]. The associated permittivity of E. coli is *ε*_*m*_ = 2.5 − 3.0 and S. cerevisae *ε*_*m*_ = 3.2 − 4.2. These are in excellent agreement with the experimentally measured results of E. coli and S. cerevisae cell membranes [68].

Comparing the current response of the 1% and 10% tethered DphPC, E. coli, and S. cerevisae membranes we see that the resistance to membrane defects from highest to lowest is: DphPC, E. coli, and S. cerevisae. As expected the resistance to membrane defects increases as the tether density increases. The difference in the resistance to membrane defects of DphPC compared to that of E. coli and S. cerevisae is a result of the phytanyl chain packing properties and the either linking the phytanyl to the lipid headgroup. The DphPC and GDEP ether-bound lipids found in archaebacterial membranes are known to have a lower lateral diffusion coefficient compared to common phospholipids found in prokaryotes and eukaryotic membranes [69]. It is suggested in [70] that the stability of the DphPC membrane is closely related to the slow conformational motion of the phytanyl chains. Therefore we conclude that the difference in the resistance to membrane defects between the DphPC, and E. coli and S. cerevisae membranes is a result of the unique dynamics of the ether-phytanyl group which may result from a hydrogen bonding difference. In addition, using the CGMD model the conformational motion of the phytanyl tail in the DphPC decreases as the tether density increases which results in the larger tether density membrane having a higher resistance to membrane defects. A surprising observation is that the E. coli membrane is more resistant to membrane defects compared to that of S. cerevisae even though the thickness of S. cerevisae is larger then that of E. coli. A possible mechanism for this difference is that defects in the E. coli membrane are primarily formed by the flip-flop of specific groups of phospholipids [66].

## Discussion

Using the CGMD model and experimental measurements from tethered bilayer lipid membranes, we summarize several key findings on how tethers and the bioelectronic gold interface impact the tethered membrane dynamics. First, in contrast to previous molecular dynamics results [71], no anomalous diffusion was detected at the surface of the membrane or in the tethering reservoir between the gold and membrane surfaces. This allows the transport of ions away from the electrode surface to be modeled using continuum theories that do not include fractional order operators [13]. For example this validates the use of the Generalized Poisson-Nernst-Planck model for computing the aqueous pore conductance in a tethered membrane as done in [6]. Second, the diffusion tensor of water and particle density are both spatially dependent. Third, the membrane thickness, lipid diffusion adjacent to the tethering reservoir, defect density, the free energy barrier for lipid flip-flop, and effective dielectric permittivity of the membrane are dependent on the tether density. This suggests that the membrane dynamics are strongly dependent on the density of cytoskeletal supports in the membrane. Fourth, experimental measurements show that a combination of diffusion-limited charge transfer and ionic adsorption are present at the surface of the gold electrodes. These double-layer charging effects can be modeled using fractional order operators [13]. Fifth, the numerical results from the CGMD model are in agreement with the results from experimental measurements from tethered bilayer lipid membranes composed of DphPC, E. coli, and S. cerevisae lipids. This supports the use of molecular dynamics to go from structure to function. All these results provide valuable insights into the dynamic properties of membranes, and into the diffusive transport of lipids and ions in natural membranes that contain cytoskeletal supports. They also yield design rules for future tethered membrane based biosensors.

## Supporting Information

S1 Text Fractional Order Macroscopic Model. The fractional order macroscopic model is used to predict important biological parameters (i.e. line tension, surface tension, membrane capacitance, and membrane conductance) from the current response of the tethered bilayer lipid membrane. The macroscopic model is constructed by making asymptotic approximations to the Smoluchowski-Einstein equation [6, 72–74] and coupling the result with a system of nonlinear fractional order differential equations that model the surface and electrolyte dynamics. The fractional order operator is included to model diffusion limited process as the bioelectronic interface which include diffusion-limited charge transfer and ionic adsorption [38].
(PDF)

S1 Table Fractional Order Macroscopic Model Parameters. The parameters *G*_*o*_, *C*_*m*_, *C*_*dl*_, and *R*_*e*_ are estimated using a single impedance measurement for each tethered membrane. The parameters *C*, *D*, *r*_*m*_, *G*_*p*_ and *W*_*es*_ in the Smoluchowski-Einstein equation are obtained from [6, 74–78]. The parameters *σ* and *γ* are computed from the CGMD simulations. Since *α* and *q* are not dependent on the tether density, only a single current measurement was used to estimate these parameters, and found to be consistent with those reported in [75].
(PDF)

S1 Fig Quality of Tethered Membrane via Impedance Measurements. Membrane defects including patches with the gold electrode directly exposed to the bulk electrolyte, portions of bilayer sandwiched together, and electrodesorption of the tethers and spacers can be detected by comparing the computed impedance to the experimentally measured impedance from the tethered bilayer lipid membrane. The experimentally measured impedance (gray dots) is in excellent agreement to the numerically computed impedance for all tether densities and membrane compositions. The phase is represented by ∠*Z*(*f*) in degrees and magnitude by *Z*(*f*). Note that Cell 1 and Cell 2 indicate the variation in impedance between tethered membranes constructed using an identical formation process.
(PDF)

S2 Fig Numerically computed slope of the round-trip time *τ*_rt_(z). The estimated slope of the round-trip time *τ*_*rt*_(*z*) is numerically computed from the CGMD bead trajectories.
(PDF)

S3 Fig Normalized water density and perpendicular diffusion *D*_⊥_(*z*) for substrate-water. The normalized density *ρ*(*z*)/*ρ*_*o*_ (a) and perpendicular diffusion coefficient *D*_⊥_(*z*) (b) are computed from the CGMD bead trajectories at the gold-interface with no membrane present.
(PDF)
