# Supplementary material for: The Effect of Tethers on Artificial Cell Membranes: A Coarse-Grained Molecular Dynamics Study
Source: PLoS One. 2016 Oct 13;11(10):e0162790. doi: 10.1371/journal.pone.0162790 (PMC5063460; doi:10.1371/journal.pone.0162790)
Supplement: S1 Text — The fractional order macroscopic model is used to predict important biological parameters (i.e. line tension, surface tension, membrane capacitance, and membrane conductance) from the current response of the tethered bilayer lipid membrane. The macroscopic model is constructed by making asymptotic approximations to the Smoluchowski-Einstein equation [6, 72–74] and coupling the result with a system of nonlinear fractional order differential equations that model the surface and electrolyte dynamics. The fractional order operator is included to model diffusion limited process as the bioelectronic interface which include diffusion-limited charge transfer and ionic adsorption [38]. (PDF) [file pone.0162790.s001.pdf]

Text S1: Fractional Order Macroscopic Model of Tethered Bilayer Lipid Membrane

The fractional order macroscopic model is used to predict important biological parameters (i.e. line tension, surface tension, membrane capacitance, and membrane conductance) from the current response of the tethered bilayer lipid membrane. The macroscopic model is constructed by making asymptotic approximations to the Smoluchowski-Einstein equation 6, 72-74 and coupling the result with a system of nonlinear fractional order differential equations that model the surface and electrolyte dynamics.

At the electrode to electrolyte interface there exists electrical double layers. For a planar electrode with no defects or tethers, this interface can be modeled using an ideal capacitor. However, as a result of the electrode surface containing minor defects from the manufacturing process, and the spacers and tethers bound to the electrode surface, there may exist diffusion-limited processes including: charge transfer and adsorption 38. In the macroscopic model, these processes are modeled using a fractional capacitor (i.e. the capacitor voltage is related, by a fractional order differential operator, to the current traveling through the capacitor). C_{tdl}, and C_{bdl} denote the fractional order capacitances for the counter electrode and electrode respectively. The bulk electrolyte solution is modeled as completely ohmic with resistance R_e. The charging dynamics of the membrane is modeled by the capacitance C_m. The tethered membrane conductance G_m is both time and membrane voltage dependent as a result of the formation and destruction of aqueous pores. The excitation potential $V_s(t)$ applied across the two electrodes closes the circuit. The governing equations of the macroscopic model of the tethered membrane are given by:

$$\begin{aligned}
\frac{dV_m}{dt} &= -(\frac{1}{C_m R_e} + \frac{G_m}{C_m})V_m - \frac{1}{C_m R_e}V_{dl} + \frac{1}{C_m R_e}V_s,\\
\frac{d^p V_{dl}}{dt^p} &= -\frac{1}{C_{dl} R_e}V_m - \frac{1}{C_{dl} R_e}V_{dl} + \frac{1}{C_{dl} R_e}V_s,\\
I(t) &= \frac{1}{R_e}\big(V_s - V_m - V_{dl}\big),
\end{aligned} \tag{S1}$$

where C_{dl} is the total capacitance of C_{tdl} and C_{bdl} in series with p denoting the order of the fractional order operator, V_m is the transmembrane potential, and V_{dl} is the double-layer potential. Given the drive potential $V_s(t)$, and the static circuit parameters C_{tdl}, C_{bdl}, C_m, and electrolyte resistance R_e, the membrane conductance G_m can be estimated from the measured current $I(t)$. For a sinusoidal excitation potential with magnitude below 50 mV and frequency f, the impedance of the tethered membrane is given by:

$$Z(f) = R_e + \frac{1}{G_m + j2\pi f C_m} + \frac{1}{(j2\pi f)^p C_{dl}}. \tag{S2}$$

with j denoting $\sqrt{-1}$. For a membrane containing negligible defects the typical values for membrane capacitance and conductance are 0.5-1.3 μF/cm^2 and 0.5-2.0 μS for an intact 1%-100% tethered membrane with surface area 2.1 mm^2–the surface area of the tethered membrane. Note that if an excitation potential above 50 mV is used then G_m will vary as a result of the formation and destruction of aqueous pores in the tethered membrane. The dynamics of G_m in (S1) can be modeled using asymptotic approximations to the Smoluchowski-Einstein as illustrated in 6.

The coupled nonlinear fractional order differential equation (S1) are numerically solved using the method presented in 6.The numerical parameters for the fractional order model are provided in the Supplementary Information S1.
