# Supplementary material for: The Effect of Tethers on Artificial Cell Membranes: A Coarse-Grained Molecular Dynamics Study
Source: PLoS One. 2016 Oct 13;11(10):e0162790. doi: 10.1371/journal.pone.0162790 (PMC5063460; doi:10.1371/journal.pone.0162790)
Supplement: S1 Table — The parameters Go, Cm, Cdl, and Re are estimated using a single impedance measurement for each tethered membrane. The parameters C, D, rm, Gp and Wes in the Smoluchowski-Einstein equation are obtained from [6, 74–78]. The parameters σ and γ are computed from the CGMD simulations. Since α and q are not dependent on the tether density, only a single current measurement was used to estimate these parameters, and found to be consistent with those reported in [75]. (PDF) [file pone.0162790.s002.pdf]

Symbol	Definition	Value		
γ	Edge energy	1.2×10^{-11} J/m		
σ	Surface tension	15×10^{-3} J/m^2		
α	Creation rate coefficient	1×10^{9} s^{-1}		
q	$q = (r_m/r_*)^2$	2.46		
C	Steric repulsion constant	9.67×10^{-15} J$^{1/4}$ m		
D	Radial diffusion coefficient	1×10^{-14} m^2/s		
r_m	Equilibrium pore radius	0.8 nm		
$G_p(r_m)$	Equilibrium pore conductance	1.56 nS		
R_e	Electrolyte resistance	100-800 Ω		
**Spacer Surface:**				
p	Fractional order parameter	0.83		
C_{dl}	Double-layer capacitance	230 nF		
**DphPC Membrane**	**Tether Density:**	**1%**	**10%**	**100%**
G_0	Initial membrane conductance	1.00 μS	0.66 μS	0.33 μS
C_m	Membrane capacitance	16.0-17.5 nF	12.5-16.0 nF	12.4 nF
p	Fractional order parameter	0.90-0.95	0.90-0.95	0.93
C_{dl}	Double-layer capacitance	100-180 nF	100-180 nF	120-180 nF
V_{ep}	Voltage of electroporation	350-415 mV	480-560 mV	650 mV
K_t	Spring constant	0 N/m	0 N/m	20 mN/m
**S. cerevisiae Membrane**	**Tether Density:**	**1%**	**10%**	
G_0	Initial membrane conductance	5.00 μS	1.11-1.66 μS	
C_m	Membrane capacitance	16.0-18.0 nF	14.0 nF	
p	Fractional order parameter	0.90	0.90-0.92	
C_{dl}	Double-layer capacitance	180 nF	180 nF	
V_{ep}	Voltage of electroporation	330-350 mV	410-430 mV	
K_t	Spring constant	0 N/m	0 N/m	
**E. coli Membrane**	**Tether Density:**	**1%**	**10%**	
G_0	Initial membrane conductance	2.00-1.00 μS	0.66 μS	
C_m	Membrane capacitance	14.0 nF	15.0-17.0 nF	
p	Fractional order parameter	0.90-0.91	0.90-0.91	
C_{dl}	Double-layer capacitance	180 nF	180 nF	
V_{ep}	Voltage of electroporation	360-380 mV	400-450 mV	
K_t	Spring constant	0 N/m	0 N/m	

Table S1: Parameters for CED Current Predictions. The parameters G_o, C_m, C_{dl}, and R_e in Table S1 are estimated using a single impedance measurement for each tethered membrane. The electroporation parameters C, D, r_m are obtained from 6, 74-78. The parameters σ and γ are computed from the CGMD simulations. Since α and q are not dependent on the tether density, only a single current measurement was used to estimate these parameters, and found to be consistent with those reported in 75.
