# Supplementary figures and images for: The Effect of Tethers on Artificial Cell Membranes: A Coarse-Grained Molecular Dynamics Study
Source: PLoS One. 2016 Oct 13;11(10):e0162790. doi: 10.1371/journal.pone.0162790 (PMC5063460; doi:10.1371/journal.pone.0162790)

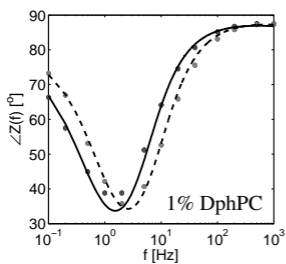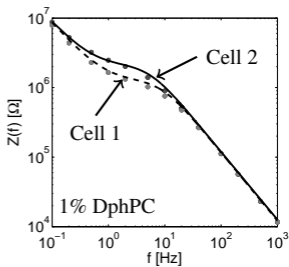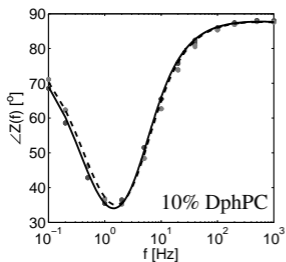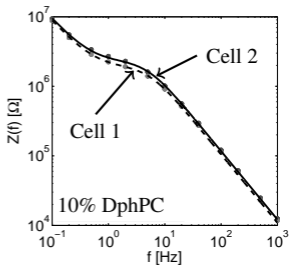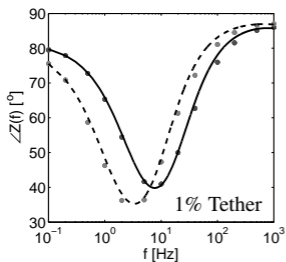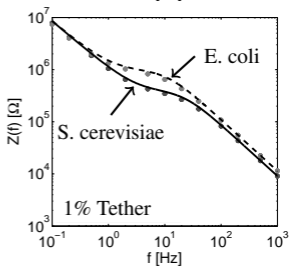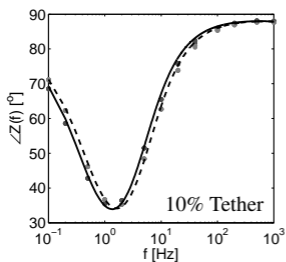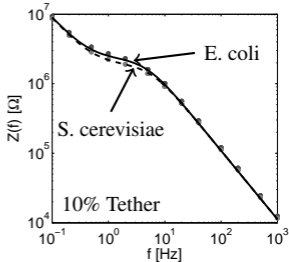

Supplement: S1 Fig — Membrane defects including patches with the gold electrode directly exposed to the bulk electrolyte, portions of bilayer sandwiched together, and electrodesorption of the tethers and spacers can be detected by comparing the computed impedance to the experimentally measured impedance from the tethered bilayer lipid membrane. The experimentally measured impedance (gray dots) is in excellent agreement to the numerically computed impedance for all tether densities and membrane compositions. The phase is represented by ∠Z(f) in degrees and magnitude by Z(f). Note that Cell 1 and Cell 2 indicate the variation in impedance between tethered membranes constructed using an identical formation process. (PDF) [file pone.0162790.s003.pdf]

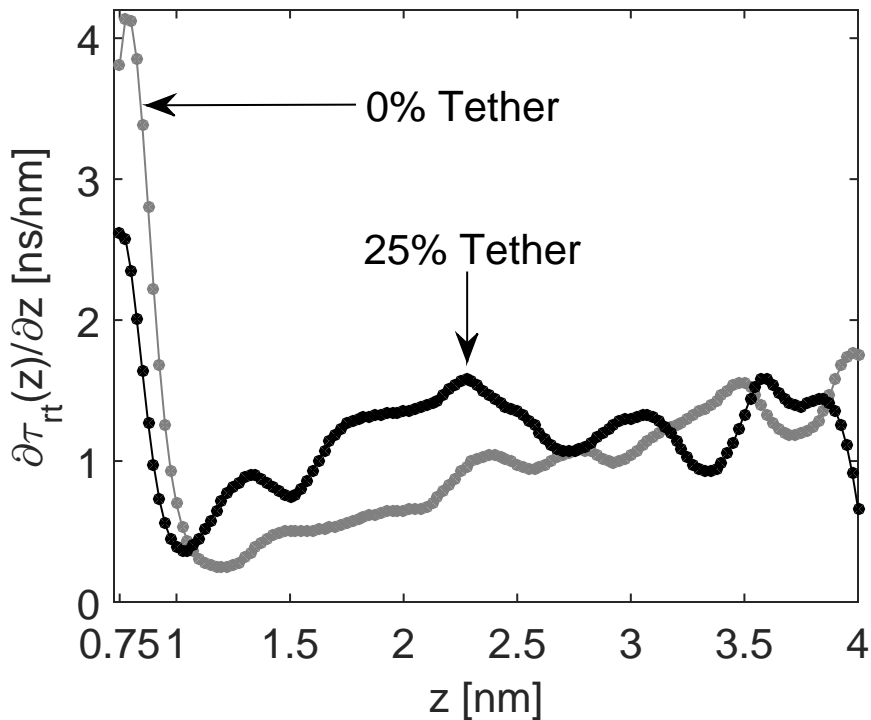

Supplement: S2 Fig — The estimated slope of the round-trip time τrt(z) is numerically computed from the CGMD bead trajectories. (PDF) [file pone.0162790.s004.pdf]

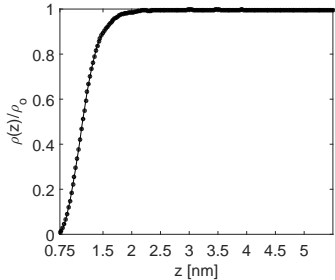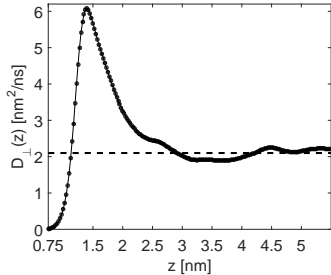

Supplement: S3 Fig — The normalized density ρ(z)/ρo (a) and perpendicular diffusion coefficient D⊥(z) (b) are computed from the CGMD bead trajectories at the gold-interface with no membrane present. (PDF) [file pone.0162790.s005.pdf]
